# The global burden of maternal disorders attributable to iron deficiency related sub-disorders in 204 countries and territories: an analysis for the Global Burden of Disease study

**DOI:** 10.3389/fpubh.2024.1406549

**Published:** 2024-09-05

**Authors:** Nuer Wu, Erdengqieqieke Ye, Yulan Ba, Shareli Caikai, Bayinsilema Ba, Ling Li, Qiying Zhu

**Affiliations:** ^1^Department of Obstetrics, Center of Maternal-Fetal Medicine, The First Affiliated Hospital of Xinjiang Medical University, Urumqi, China; ^2^Department of Prenatal Diagnosis, Reproductive Medicine Center, The First Affiliated Hospital of Xinjiang Medical University, Urumqi, China; ^3^Department of Rehabilitation Medicine, People’s Hospital of Xinjiang Uygur Autonomous Region, Urumqi, China; ^4^Department of Respiratory Intensive Care Unit, The First Affiliated Hospital of Xinjiang Medical University, Urumqi, China; ^5^Department of Cardiology, The Fifth Affiliated Hospital of Xinjiang Medical University, Urumqi, China

**Keywords:** GBD 2019, maternal disorders, iron deficiency, Disability-Adjusted Life Years, Estimated Annual Percentage Changes

## Abstract

**Background:**

Pregnancy-related anemia presents a significant health concern for approximately 500 million women of reproductive age worldwide. To better prevent maternal disorders, it is essential to understand the impact of iron deficiency across different maternal disorders, regions, age groups, and subcategories.

**Methods:**

Based on the comprehensive maternal disorders data sourced from the 2019 Global Burden of Disease study, an investigation was carried out focusing on Disability-Adjusted Life Years (DALYs) associated with iron deficiency spanning the period from 1990 to 2019. In addition, Estimated Annual Percentage Changes (EAPCs) were computed for the duration of the study.

**Results:**

Our study indicates decreasing mortality rates and years of life lost due to maternal conditions related to iron deficiency, such as maternal hemorrhage, miscarriage, abortion, hypertensive disorders, and infections. However, mortality rates and years of life lost due to indirect and late maternal deaths, as well as deaths aggravated by HIV/AIDS, have increased in high socio-demographic index (SDI) regions, especially in North America. Moreover, the proportion of maternal deaths aggravated by HIV/AIDS due to iron deficiency is rising globally, especially in Southern Sub-Saharan Africa, Oceania, and Georgia. In addition, in the Maldives, the age-standardized DALYs for maternal disorders attributable to iron deficiency exhibited a notable decreasing trend, encompassing a range of conditions. Furthermore, there was a significant decrease in Disability-Adjusted Life Years rate for miscarriages and preterm births among women aged 15–49, with hypertensive disorders posing the highest burden among women aged 15–39.

**Conclusion:**

The burden of maternal disorders caused by iron deficiency is decreasing in most regions and subtypes, except for deaths aggravated by HIV/AIDS. By thoroughly understanding the details of how iron deficiency impacts the health of pregnant women, health policymakers, healthcare professionals, and researchers can more effectively pinpoint and address the root causes of inequalities in maternal health.

## 1 Introduction

According to data from the World Health Organization (WHO) data in 2017, around 211 mothers died for every 100,000 live births in 2017, totaling roughly 295,000 maternal deaths (1). Additionally, about 15% of pregnant women face serious pregnancy complications each year (2). These complications not only endanger the health of mothers but also affect the well-being of babies (2, 3). By 2015, global maternal mortality dropped by 44%, missing the 75% reduction target. Maternal mortality in low-income countries remained nearly 20 times higher than in high-income countries (4).

The WHO has previously esmated that anemia is responsible for a significant global burden of deaths among women of reproductive age, with an estimated 16,800 to 28,000 deaths occurring annually (5). A 2016 report from the WHO underscored that more than 40% of expectant mothers encountered anemia (6, 7). Anemia not only affects the health of newborns, including increasing the risk of preterm birth, low birth weight, and fetal and neonatal mortality but also increases the risk of maternal mortality during childbirth and the postpartum period (8). Severe anemia may lead to ineffective compensation by the mother’s body, increasing cardiac workload and the risk of bleeding, reducing tolerance to blood loss, and potentially leading to circulatory shock and death (9). A study revealed a linear relationship between pregnancy-related anemia and mortality, indicating that for every 10 g/L increase in hemoglobin, there was a 29% decrease in maternal mortality among pregnant and postpartum women (10). Another study, the largest-scale investigation of severe obstetric complications covering 29 countries, concluded that the occurrence of severe anemia during pregnancy or postpartum (defined as hemoglobin concentration below 70 g/L) doubles the risk of maternal mortality in this population of pregnant and postpartum women (11). Additional literature suggests that around 20% of maternal fatalities stem from anemia, with this condition serving as an additional contributing risk factor in half of all maternal deaths (12). The most common type of anemia is iron deficiency, which is a condition resulting from a lack of proper nutrition (13, 14). According to the Global Burden of Diseases, Injuries, and Risk Factors Study (GBD) 2019, iron deficiency has been identified as the primary risk factor contributing to the burden of maternal disorders. The GBD 2019 provides a standardized and comprehensive assessment of risk factor exposure, relative risk, and disease burden (15). The Global Burden of Disease (GBD) study is a collaborative effort involving multiple international institutions, led by the Institute for Health Metrics and Evaluation (IHME) at the University of Washington. The study aims to provide health data at global, regional, and national levels to assist in policy-making and the implementation of health interventions (16).

Although, some studies have investigated the global burden of maternal disorders and the inequality surrounding iron deficiency (17–22). Those studies did not further analyze the burden of iron deficiency in relation to ectopic pregnancy, indirect maternal deaths, late maternal deaths, maternal hemorrhage, maternal hypertensive disorders, maternal obstructed labor and uterine rupture, maternal sepsis and other maternal infections, other maternal disorders. Understanding the burden of iron deficiency across different maternal disorders, regions, age groups, and subcategories is crucial for more accurate prevention of maternal disorders.

Therefore, we conducted an analysis utilizing data from the GBD 2019 to ascertain the worldwide, regional, and national age-standardized rates (ASRs) of death and disability-adjusted life-years (DALYs) for 10 different maternal disorders due to iron deficiency from 1990 to 2019. This analysis was conducted in terms of age-standardized rates spanning from 1990 to 2019, considering various demographic factors such as age, geographical regions, countries, and the Sociodemographic Index (SDI). The objective was to offer a comprehensive and comparable examination of the burden of iron deficiencies among these 10 maternal disorders.

## 2 Methods

### 2.1 Data source

The data on maternal disorders caused by iron deficiency were sourced from the Global Health Data Exchange (GHDx) using the GBD Results Tool.^1^ GHDx, managed by IHME, offers extensive global health data, including disease burden, health risk factors, and healthcare utilization across multiple countries and regions, providing a comprehensive global health perspective. The data on GHDx undergoes rigorous quality control and verification to ensure accuracy and consistency, with standardized formats for cross-national comparisons. Additionally, GHDx provides detailed metadata, clarifying data sources, collection methods, and limitations, aiding in the correct interpretation and use of the data (23, 24). The GBD 2019 study estimated 369 diseases and injuries, as well as 87 risk factors, across 204 countries and territories. Detailed information on the original data and general methodology of the GBD 2019 study has been previously published (15). In this study, we collected data for 10 different maternal disorders due to iron deficiency from 1990 to 2019, along with their 95% uncertainty intervals (UI). We also gathered sex, age, Socio-Demographic Index (SDI), and geographic location information to conduct a more comprehensive analysis of the disease burden. Additionally, we obtained the DALY rate of maternal disorders due to iron deficiency categorized by age group.

### 2.2 Definition

In GBD 2019, maternal disorders only have one specific risk factor: Iron deficiency. The WHO reports that anemia results in 16,800 to 28,000 deaths annually among reproductive-age women (25). Iron deficiency anemia (IDA) complicates 30–60% of pregnancies globally, with nearly 75% of pregnant women affected by the third trimester (26–28). Iron deficiency is a key factor in maternal mortality, highlighted as the primary risk factor for maternal disorders in the GBD 2019 study.

Iron deficiency is characterized by insufficient iron levels to meet the body’s requirements. Dietary iron deficiency is defined as having an inadequate amount of dietary iron to meet the body’s needs due to insufficient intake of iron from the diet, rather than being caused by other absolute or functional deficiencies in iron (29). Age-standardized rates (ASRs) measure per 100,000 people, adjusted for standard age composition to allow fair regional comparisons. This adjustment eliminates the impact of varying age structures, making statistical indicators comparable (19). Disability-Adjusted Life Years (DALYs) is a metric used to measure the burden of disease. It takes into account the years of life lost due to premature death (YLL) and the years lived with disability or poor health (YLD) (30). YLL refers to the years of life lost due to premature death, while YLD refers to the years lived with disability or poor health (20). The Socio-demographic Index (SDI) is designed to measure and compare levels of socio-economic development among different regions, countries, or populations. The 204 countries and territories are divided by SDI quintiles into five regions (low SDI, low-middle SDI, middle SDI, middle-high SDI, and high SDI), and geographically into 21 GBD regions (29, 31).

The causes of maternal disorders were classified according to the categories listed in the 2019 GBD study (15). The operational definition based on the International Classification of Diseases-10th Revision (ICD-10) are list on Supplementary Table S1.

### 2.3 Statistical analysis

To understand the temporal trends in death and Disability-Adjusted Life Years (DALYs), the Estimated Annual Percentage Changes (EAPC) is calculated. EAPC is derived by conducting a log-linear regression on calendar years, by fitting the model ln(y) = α + βx + ε to the natural logarithm of the rates, where y represents age-standardized rates, and x represents calendar years. The exponentiated value of the regression model’s slope coefficient, exp(β), is utilized. EAPC is expressed as a percentage change by subtracting 1 from exp(β) and then multiplying the result by 100. The 95% confidence intervals (CI) for EAPC are calculated based on the linear model. This interval provides a range in which the true EAPC is expected to fall with 95% confidence. If the upper limit of the 95% CI for EAPC is less than 0, it indicates a downward trend over time; conversely, if the lower limit of the 95% CI is greater than 0, it suggests an upward trend. If neither condition is met, it suggests a stable trend, indicating that rates remain relatively consistent during the given period. Statistical analyses and the visualization of results were conducted using the R software (version 4.3.1, R Core Team).

## 3 Results

### 3.1 Overall impact of iron deficiency on maternal disorder burden

**Figure 1:**
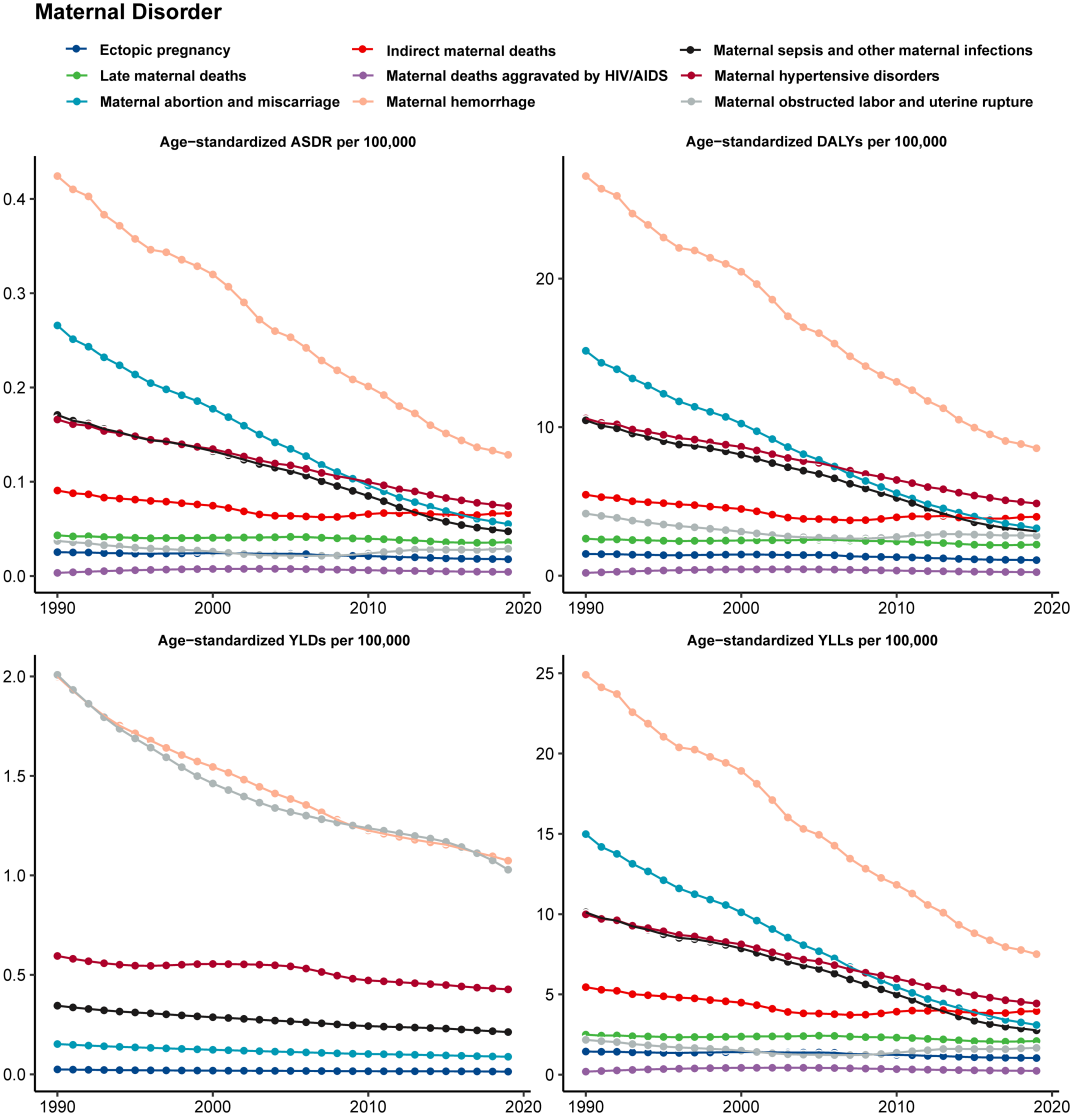
The temporal trends in the burden of iron deficiency on maternal disorders from 1990 to 2019.

Global data from 1990 to 2019 shows clear downward trends in the EAPC of the Age Standardized Death Rate (ASDR), age-standardized DALYs, and age-standardized YLLs for maternal hemorrhage, maternal abortion and miscarriage, maternal hypertensive disorders, and maternal sepsis and other maternal infections due to iron deficiency (Figure 1). The other five maternal disorders showed steady trends (Figure 1). As for age-standardized years YLD, except for maternal obstructed labor and uterine rupture and maternal hemorrhage, which showed a significant decrease, the others either lack data or remain stable (Figure 1). The most pronounced declines in disease burden were observed in maternal hemorrhage.

From 1990 to 2019, the high SDI region exhibited an increasing trend in age-standardized DALYs due to indirect maternal deaths, late maternal deaths, and maternal deaths aggravated by HIV/AIDS related to iron deficiency, with the most pronounced increase observed in high-income North America. Similarly, maternal deaths aggravated by HIV/AIDS due to iron deficiency also rose in the Middle SDI region. However, all other categories showed a decrease or stable (Table 1). Within the 21 GBD regions, a significant decline in age-standardized DALYs was observed for iron deficiency-associated maternal abortion and miscarriage in Central Europe (EAPC = −10.88 [−11.53–10.23]). Similarly, a notable decrease was seen in iron deficiency-related maternal hemorrhage in East Asia (EAPC = −10.6 [−11.14–10.07]). In contrast, Oceania experienced the sharpest uptick in age-standardized DALYs for maternal deaths aggravated by HIV/AIDS due to iron deficiency, marking the most considerable increasing trend at an EAPC of 15.13 (95% UI: 10.81–19.62) as detailed in Table 1.

**Table 1:** The EAPC of age-standardized rates of DALY burden caused by maternal disorders from iron deficiency from 1990 to 2019.

Characteristics	Ectopic pregnancy	Indirect maternal deaths	Late maternal deaths	Maternal abortion and miscarriage	Maternal deaths aggravated by HIV/AIDS
Global	−1.13 (−1.32–0.95)	−1.17 (−1.42–0.91)	−0.54 (−0.67–0.41)	−5.38 (−5.6–5.16)	−0.42 (−1.38–0.54)
Low SDI	−1.48 (−1.64–1.32)	−0.5 (−0.7–0.3)	−1.04 (−1.26–0.81)	−5.15 (−5.5–4.81)	−2.71 (−3.51–1.9)
Low-middle SDI	−2.12 (−2.38–1.86)	−2.28 (−2.68–1.87)	−2.2 (−2.28–2.13)	−8.88 (−9.24–8.53)	0.75 (−0.31–1.82)
Middle SDI	−3.33 (−3.74–2.92)	−2.36 (−2.56–2.16)	−1.03 (−1.25–0.82)	−9.2 (−9.44–8.96)	4.39 (1.66–7.18)
High-middle SDI	−5.16 (−5.4–4.91)	−3.94 (−4.25–3.63)	−1.97 (−2.3–1.63)	−8.07 (−8.47–7.67)	−1.14 (−3.16–0.92)
High SDI	−2.89 (−3.05–2.74)	3.4 (2.86–3.94)	3.22 (2.57–3.87)	−4.93 (−5.18–4.68)	4.45 (3.97–4.93)
East Asia	−6.89 (−7.35–6.44)	−6.74 (−7.54–5.92)	−7.75 (−8.19–7.31)	−9.04 (−9.41–8.67)	−2.5 (−3.85–1.13)
Southeast Asia	−3.4 (−3.65–3.15)	−1.5 (−1.6–1.39)	−3.53 (−3.65–3.41)	−9.91 (−10.12–9.71)	0.69 (−1.66–3.1)
Central Asia	−2.44 (−2.59–2.29)	1.46 (0.8–2.13)	−2.04 (−2.36–1.73)	−8.64 (−9.01–8.28)	4.18 (3.78–4.57)
South Asia	−3.37 (−3.68–3.05)	−3.27 (−3.74–2.8)	−3.48 (−3.63–3.33)	−10.3 (−10.93–9.67)	1.35 (−1.99–4.8)
Australasia	−3.62 (−4.29–2.94)	0.55 (−0.32–1.41)	−2.54 (−3.31–1.78)	−2.16 (−2.46–1.86)	4 (3.28–4.72)
Oceania	−0.77 (−1.05–0.5)	0.33 (0–0.65)	−1.08 (−1.23–0.92)	−4.89 (−5.09–4.69)	15.13 (10.81–19.62)
Central Europe	−6.36 (−6.96–5.76)	0.17 (−1.3–1.66)	−4.14 (−4.62–3.64)	−10.88 (−11.53–10.23)	2.31 (1.64–2.99)
Eastern Europe	−8.14 (−8.51–7.77)	−4.48 (−4.76–4.2)	−4.77 (−5.12–4.43)	−6.62 (−6.85–6.39)	9.4 (8.97–9.84)
Western Europe	−3.4 (−3.55–3.25)	−2.55 (−2.91–2.19)	−3.9 (−4.24–3.55)	−3.77 (−4.15–3.39)	−0.09 (−0.52–0.33)
High-income Asia Pacific	−5.36 (−5.61–5.1)	−3.53 (−3.8–3.25)	−3.17 (−3.69–2.65)	−8.45 (−9.1–7.79)	2.85 (2.57–3.12)
High-income North America	−2.32 (−2.61–2.03)	8.06 (7.01–9.12)	4.16 (3.37–4.95)	−2.29 (−2.51–2.06)	5.42 (4.86–5.99)
Southern Latin America	−0.42 (−0.73–0.11)	2.7 (2.07–3.34)	0.78 (0.47–1.09)	−4.69 (−4.88–4.49)	2.48 (1.66–3.3)
Andean Latin America	0.72 (−0.17–1.62)	3.4 (2.82–3.98)	−4.19 (−4.57–3.8)	−9.77 (−10.32–9.21)	−0.22 (−0.63–0.2)
Central Latin America	−3.11 (−3.42–2.79)	1.38 (1–1.76)	−1.19 (−1.96–0.41)	−7.26 (−7.68–6.85)	2.45 (1.9–3)
Tropical Latin America	−3.14 (−3.89–2.39)	0.06 (−0.12–0.25)	−1.37 (−2.36–0.38)	−5.58 (−5.99–5.17)	2.24 (1.94–2.54)
Caribbean	5.81 (4.89–6.74)	2.34 (1.96–2.72)	1.28 (1.07–1.5)	−3.44 (−3.68–3.19)	−1.15 (−1.52–0.77)
North Africa and Middle East	−3.5 (−3.57–3.44)	−1.21 (−1.26–1.15)	−2.67 (−2.75–2.58)	−7.2 (−7.35–7.05)	1.05 (−0.08–2.18)
Central Sub-Saharan Africa	−0.73 (−1.04–0.41)	0.98 (0.61–1.35)	−0.3 (−0.76–0.16)	−4.47 (−4.85–4.09)	−2.32 (−3.3–1.33)
Eastern Sub-Saharan Africa	−2.19 (−2.27–2.11)	−0.91 (−1.26–0.55)	−2.02 (−2.15–1.9)	−6.79 (−7.14–6.44)	−4.08 (−4.86–3.29)
Southern Sub-Saharan Africa	−2.1 (−3.29–0.89)	1.9 (0.15–3.67)	−0.9 (−2.06–0.27)	−2.68 (−4.1–1.24)	2.66 (0.59–4.78)
Western Sub-Saharan Africa	−1.07 (−1.41–0.73)	1.23 (1–1.46)	−0.01 (−0.38–0.35)	−2.86 (−3.29–2.43)	0.8 (−0.48–2.09)

Characteristics	Maternal hemorrhage	Maternal hypertensive disorders	Maternal obstructed labor and uterine rupture	Maternal sepsis and other maternal infections	Other maternal disorders
Global	−3.99 (−4.15–3.83)	−2.72 (−2.82–2.61)	−1.35 (−1.73–0.96)	−4.32 (−4.66–3.98)	−0.81 (−1.07–0.56)
Low SDI	−3.6 (−3.79–3.41)	−2.22 (−2.41–2.03)	−1.79 (−2.06–1.53)	−3.89 (−4.41–3.38)	−0.87 (−1.07–0.66)
Low-middle SDI	−5.27 (−5.49–5.05)	−4.35 (−4.51–4.19)	−2.53 (−3.04–2.02)	−7.59 (−7.97–7.2)	−1.62 (−2–1.23)
Middle SDI	−5.78 (−5.98–5.57)	−4.7 (−4.88–4.52)	−1.99 (−2.18–1.8)	−7.68 (−8–7.36)	−2.68 (−3.04–2.32)
High-middle SDI	−6.35 (−6.55–6.15)	−5.36 (−5.55–5.17)	−3.54 (−3.82–3.27)	−6.83 (−7.24–6.42)	−3.47 (−3.68–3.25)
High SDI	−2.85 (−2.94–2.76)	−1.66 (−1.77–1.56)	−1.68 (−1.86–1.5)	−2.68 (−2.94–2.41)	0.54 (0.27–0.82)
East Asia	−10.6 (−11.14–10.07)	−8.8 (−9.29–8.31)	−5.5 (−6.08–4.91)	−9.16 (−9.73–8.58)	−6.06 (−6.43–5.69)
Southeast Asia	−5.79 (−6.01–5.56)	−3.97 (−4.21–3.73)	−2.12 (−2.5–1.74)	−7.24 (−7.57–6.91)	−2.71 (−3.06–2.36)
Central Asia	−4.43 (−4.63–4.23)	−2.83 (−3.14–2.52)	−1.58 (−1.93–1.23)	−3.52 (−3.75–3.29)	−1.76 (−2.06–1.46)
South Asia	−5.84 (−6.12–5.55)	−4.76 (−4.91–4.6)	−3.94 (−4.52–3.37)	−8.63 (−9.22–8.03)	−2.57 (−3.06–2.07)
Australasia	−1.07 (−1.31–0.82)	−2.21 (−2.5–1.92)	0 (−0.13–0.13)	−0.27 (−0.48–0.06)	−0.77 (−1.27–0.27)
Oceania	−2.23 (−2.38–2.07)	−0.66 (−0.99–0.33)	−0.18 (−0.48–0.12)	−2.29 (−2.71–1.88)	−0.5 (−0.78–0.21)
Central Europe	−3.43 (−3.83–3.03)	−3.21 (−3.59–2.83)	−1.22 (−1.53–0.91)	−4.94 (−5.47–4.4)	−2 (−2.5–1.49)
Eastern Europe	−3.2 (−3.41–3)	−3.05 (−3.37–2.72)	−2.04 (−2.16–1.91)	−2.81 (−3.04–2.58)	−2.09 (−2.41–1.76)
Western Europe	−1.97 (−2.16–1.79)	−1.88 (−2.05–1.71)	−0.18 (−0.28–0.08)	−1.66 (−2–1.31)	−0.44 (−0.67–0.2)
High-income Asia Pacific	−4.33 (−4.59–4.06)	−5.6 (−6.15–5.05)	−2.14 (−2.36–1.92)	−6.63 (−7.74–5.5)	−1.98 (−2.17–1.79)
High-income North America	−0.84 (−1.01–0.66)	0.41 (0.02–0.8)	−2.02 (−2.49−−1.55)	-1.5 (−1.8–1.21)	3 (2.46–3.53)
Southern Latin America	−3.09 (−3.21–2.96)	−2.83 (−3–2.65)	−0.56 (−0.72–0.4)	−3.98 (−4.41–3.54)	2.55 (1.75–3.36)
Andean Latin America	−6.32 (−6.55–6.09)	−5.62 (−5.88–5.37)	2.21 (1.83–2.59)	−8.26 (−8.74–7.78)	1.99 (0.97–3.02)
Central Latin America	−4.73 (−4.97–4.5)	−3.83 (−4.08–3.58)	−1.67 (−1.89–1.45)	−5.97 (−6.43–5.51)	−3.29 (−3.56–3.02)
Tropical Latin America	−4.18 (−4.53–3.84)	−3.67 (−4.06–3.28)	−2.7 (−3.01–2.39)	−3.71 (−4.21–3.19)	−2.62 (−3.06–2.17)
Caribbean	0.57 (0.26–0.89)	1.34 (1.02–1.66)	3.97 (3.4–4.54)	0.67 (0.33–1.02)	6.64 (5.68–7.62)
North Africa and Middle East	−4.71 (−4.82–4.59)	−4.35 (−4.45–4.25)	−2.35 (−2.38–2.32)	−5.99 (−6.23–5.75)	−2.35 (−2.48–2.22)
Central Sub-Saharan Africa	−2.6 (−2.87–2.33)	−1.96 (−2.25–1.67)	−2.06 (−2.22–1.9)	−0.1 (−1.16–0.97)	0.44 (0.06–0.82)
Eastern Sub-Saharan Africa	−3.46 (−3.55–3.37)	−2.33 (−2.51–2.16)	−1.17 (−1.42–0.93)	−5.04 (−5.47–4.6)	−1.7 (−1.78–1.61)
Southern Sub-Saharan Africa	−2.28 (−3.36–1.19)	−1.4 (−2.59–0.18)	−1.37 (−1.91–0.82)	−0.77 (−2.09–0.56)	−0.66 (−1.83–0.52)
Western Sub-Saharan Africa	−1.88 (−2.15–1.62)	−1.29 (−1.55–1.02)	−0.01 (−0.15–0.13)	−3.42 (−3.8–3.03)	−0.49 (−0.72–0.26)

From 1990 to 2019, within the five SDI regions, the ASDR for maternal abortion and miscarriage associated with iron deficiency saw the most significant decline in the Middle SDI region, with an EAPC of −9.79 (95% UI: −10.05–9.52). Maternal deaths aggravated by HIV/AIDS increased in the Middle SDI region (EAPC = 4.52 [1.83–7.28]) and were stable in the Low-middle SDI region (EAPC = 0.97 [−0.07–2.03]), while all other categories showed a decrease. Among the maternal disorders across the 21 GBD regions, the largest increase occurred in maternal deaths aggravated by HIV/AIDS in Oceania (EAPC = 15.42 [11.17–19.83]), while the largest decrease was observed in Maternal sepsis and other maternal infections in High-income Asia Pacific (EAPC = −13.18 [−14.85–11.47]) (Supplementary Table S2).

In GBD 2019, data on the age-standardized YLDs related to maternal disorders due to iron deficiency were missing for indirect maternal deaths, late maternal deaths, and maternal deaths aggravated by HIV/AIDS. However, in five regions categorized by SDI, trends in ectopic pregnancy, maternal abortion and miscarriage, maternal hemorrhage, maternal hypertensive disorders, maternal obstructed labor and uterine rupture, and maternal sepsis and other maternal infections showed a decreasing trend. Among 21 GBD regions, the age-standardized YLDs of maternal abortion and miscarriage due to iron deficiency shows the largest decrease in High-income Asia Pacific (EACP = −7.34 [−8.25–6.42]) (Supplementary Table S3).

In the five SDI regions, the trend of age-standardized YLLs due to iron deficiency-related indirect maternal deaths, late maternal deaths, and maternal deaths aggravated by HIV/AIDS were increasing in the High SDI region. Maternal deaths aggravated by HIV/AIDS were also increasing in the Middle SDI region and remained stable in the Low-middle SDI region. In all other maternal disorders across SDI regions, there was a decrease. In 21 GBD regions, the age-standardized YLLs of maternal abortion and miscarriage caused by iron deficiency are decreasing. Central Europe shows the most significant decline with an EAPC of −12.45 (95%: −13.06–11.83), Conversely, maternal deaths aggravated by HIV/AIDS in Oceania shows the most significant increase, with an EAPC of 15.13 (95%:10.81–19.62) (Supplementary Table S4).

### 3.2 The temporal trends in DALY burden of maternal disorders caused by iron deficiency across GBD regions

**Figure 2:**
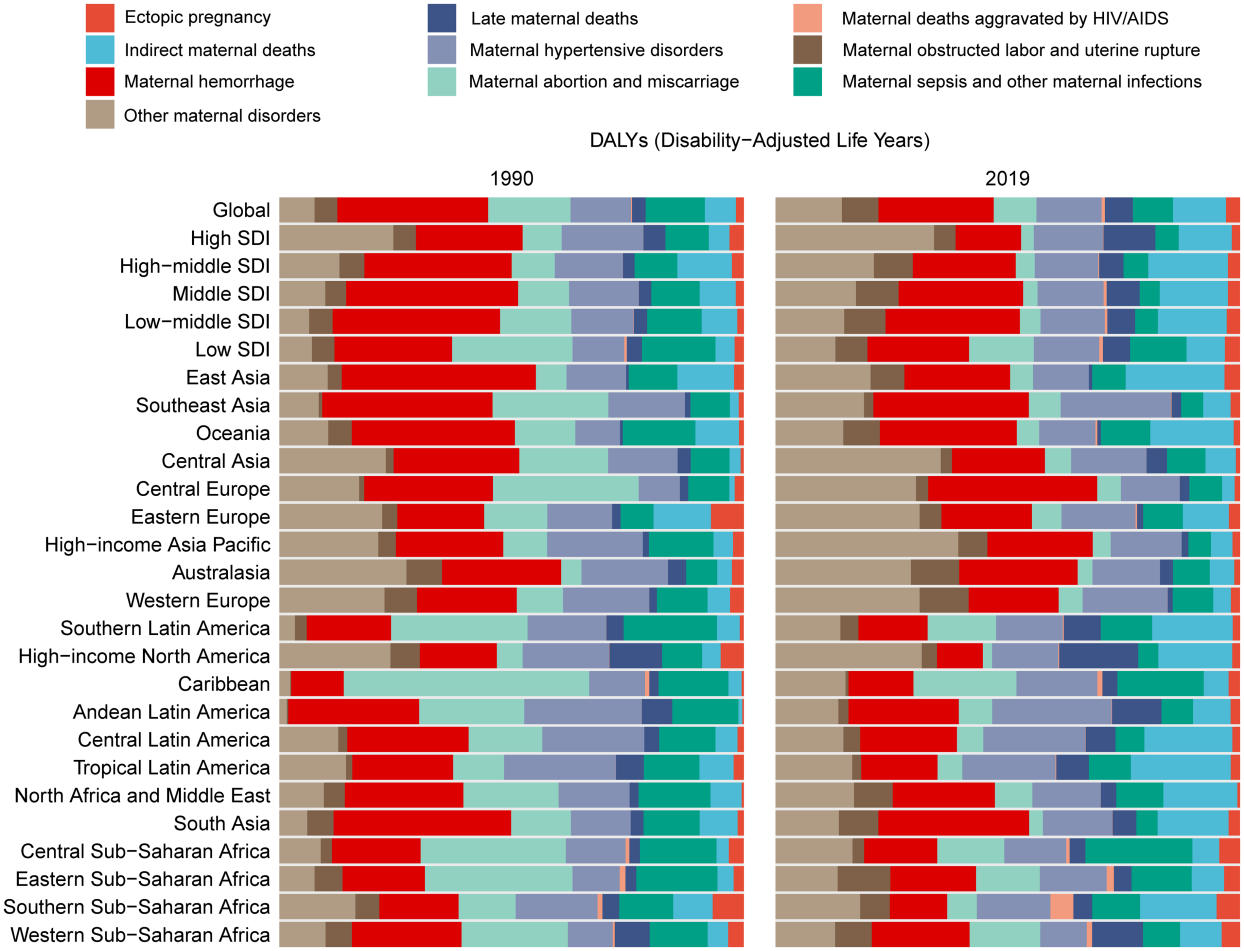
The proportion of maternal disorders contribution to iron deficiency across GBD regions between 1990 and 2019.

Between 1990 and 1999, the proportion of maternal abortion and miscarriage in the age-standardized DALYs of maternal disorders due to iron deficiency decreased across GBD regions. Meanwhile, the proportions of Indirect maternal deaths and Other maternal disorders increased (Figure 2) among most of regions. Additionally, we can see that maternal hemorrhage and Other maternal disorders account for a larger proportion of the DALYs of 10 maternal disorders associated with iron deficiency. In 1990, East Asia and South Asia had relatively higher proportions of maternal hemorrhage, but by 2019, these proportions had decreased, while in Central Europe it increased. Meanwhile, the proportion of maternal deaths aggravated by HIV/AIDS increased proportionally globally and across different SDI levels, with the most significant increase observed in Southern Sub-Saharan Africa. The proportion of late maternal deaths increased globally and across all five SDI levels, with the most significant increase observed in High-income North America.

### 3.3 The temporal trends in DALY burden of maternal disorders caused by iron deficiency across 204 countries

**Figure 3:**
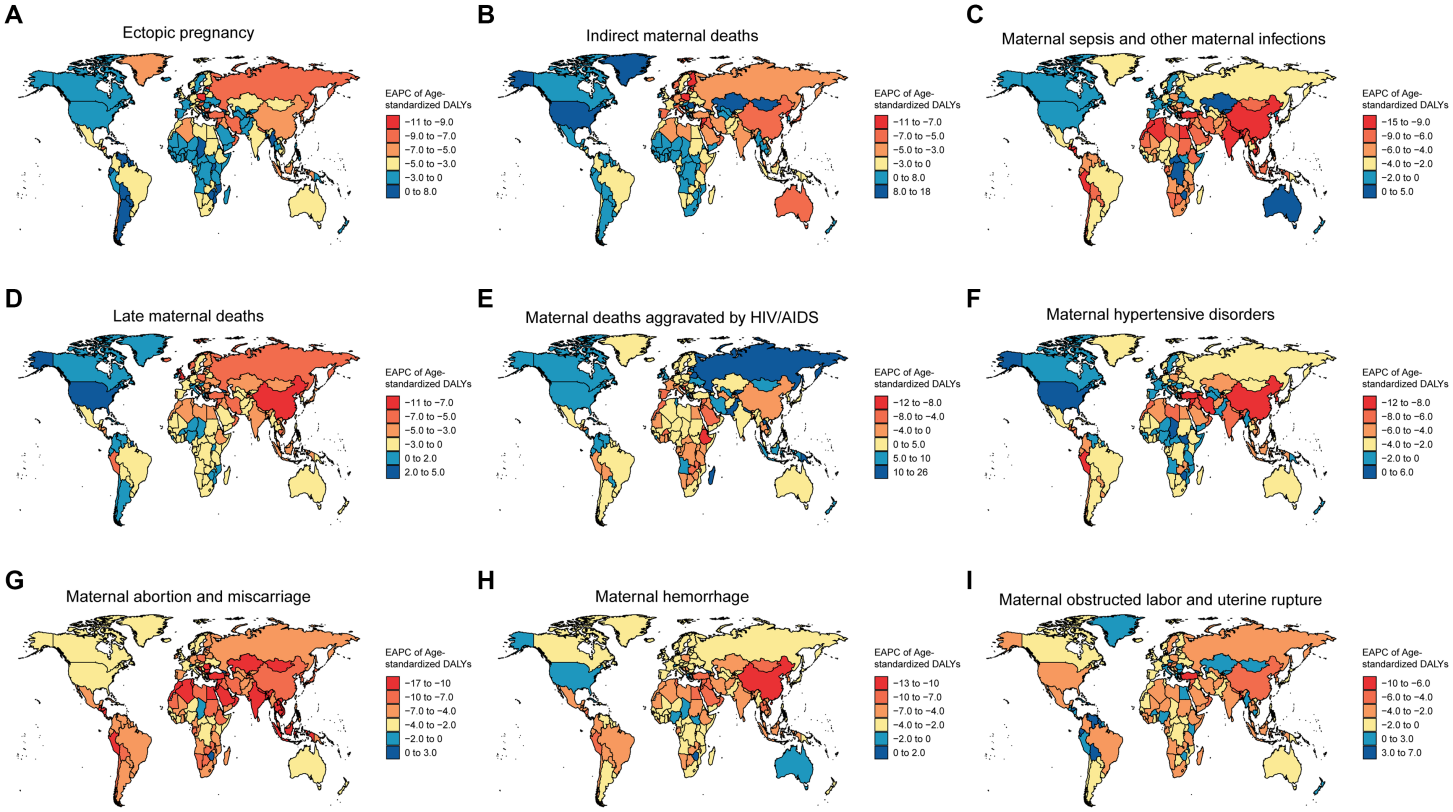
The EAPC of age-standardized DALYs for maternal disorders caused by iron deficiency across 204 countries. **(A)** The EAPC of age-standardized DALYs for ectopic pregnancy caused by iron deficiency across 204 countries. **(B)** The EAPC of age-standardized DALYs for indirect maternal deaths caused by iron deficiency across 204 countries. **(C)** The EAPC of age-standardized DALYs for maternal sepsis and other maternal infections caused by iron deficiency across 204 countries. **(D)** The EAPC of age-standardized DALYs for late maternal deaths caused by iron deficiency across 204 countries. **(E)** The EAPC of age-standardized DALYs for maternal deaths aggravated by HIV/AIDS caused by iron deficiency across 204 countries. **(F)** The EAPC of age-standardized DALYs for maternal hypertensive disorders caused by iron deficiency across 204 countries. **(G)** The EAPC of age-standardized DALYs for maternal abortion and miscarriage caused by iron deficiency across 204 countries. **(H)** The EAPC of age-standardized DALYs for maternal hemorrhage caused by iron deficiency across 204 countries. **(I)** The EAPC of age-standardized DALYs for maternal obstructed labor and uterine rupture caused by iron deficiency across 204 countries.

The EAPC, representing the average annual percentage change and direction in age-standardized DALYs for maternal disorders caused by iron deficiency across 204 countries, is presented in Figure 3. At the national level, the biggest increase in the EAPC of age-standardized DALYs is seen in Georgia due to maternal deaths aggravated by HIV/AIDS related to iron deficiency (EAPC = 25.02 [22.39–27.71]) (Figure 3E and Supplementary Table S5). The most significant decrease in maternal disorders in terms of age-standardized DALYs in the Eastern Mediterranean Region is observed in Maldives, primarily due to maternal abortion and miscarriage related to iron deficiency (EACP = −16.1 [−16.61–15.59]). This is followed by Bosnia and Herzegovina, as well as Oman (Figure 3G and Supplementary Table S5).

The age-standardized DALYs for ectopic pregnancy show an increasing trend in a few countries such as Dominica, Cuba, and Jamaica, while showing a decreasing trend in other regions, especially in Estonia, Bulgaria, Maldives, and Russia (Figure 3A and Supplementary Table S5). Indirect maternal deaths’ age-standardized DALYs demonstrate an increasing trend in some countries, notably in Greenland, United States of America, and Hungary, while witnessing a significant decrease in Cyprus, Qatar, and Finland (Figure 3B and Supplementary Table S5). Maternal sepsis and other maternal infections are primarily showing a decreasing trend across 204 countries, with Equatorial Guinea, Maldives, and Nepal being the top three countries with the most prominent decrease (Figure 3C and Supplementary Table S5). Late maternal deaths’ age-standardized DALYs show a significant decrease in Estonia, Bulgaria, and Maldives, while displaying a slight upward trend in Hungary, Dominica, and Cuba (Figure 3D and Supplementary Table S5). Similarly, maternal hypertensive disorders are generally on a downward trend, with the most significant decreases observed in Maldives, Jordan, and the Republic of Korea (Figure 3F and Supplementary Table S5). The DALYs for maternal hemorrhage is showing a decreasing trend in Maldives, China, and the Syrian Arab Republic (Figure 3H and Supplementary Table S5). maternal obstructed labor and uterine rupture are displaying an upward trend in some regions such as Venezuela, Guyana, and Honduras, while showing a downward trend in others, for example, Bangladesh, Bhutan, and Qatar (Figure 3I and Supplementary Table S5).

In the Maldives, the age-standardized DALYs for maternal disorders attributable to iron deficiency exhibited a notable decreasing trend, encompassing a range of conditions. This includes ectopic pregnancy, with an EAPC of −8.89 (95% CI: −9.3 to −8.47), Indirect maternal deaths (EAPC = −7.42 [95% CI: −7.72 to −7.13]), Late maternal deaths (EAPC = −6.45 [95% CI: −6.91 to −5.99]), Maternal abortion and miscarriage (EAPC = −16.1 [95% CI: −16.61 to −15.59]), maternal deaths aggravated by HIV/AIDS (EAPC = −6.76 [95% CI: −6.99 to −6.53]), maternal hemorrhage (EAPC = −12.76 [95% CI: −13.1 to −12.42]), maternal sepsis and other maternal infections (EAPC = −11.59 [95% CI: −12.28 to −10.9]), and Other maternal disorders (EAPC = −8.88 [95% CI: −9.27 to −8.49]) (Supplementary Table S5).

### 3.4 Iron deficiency contributions to maternal disorders DALY rates by age group

**Figure 4:**
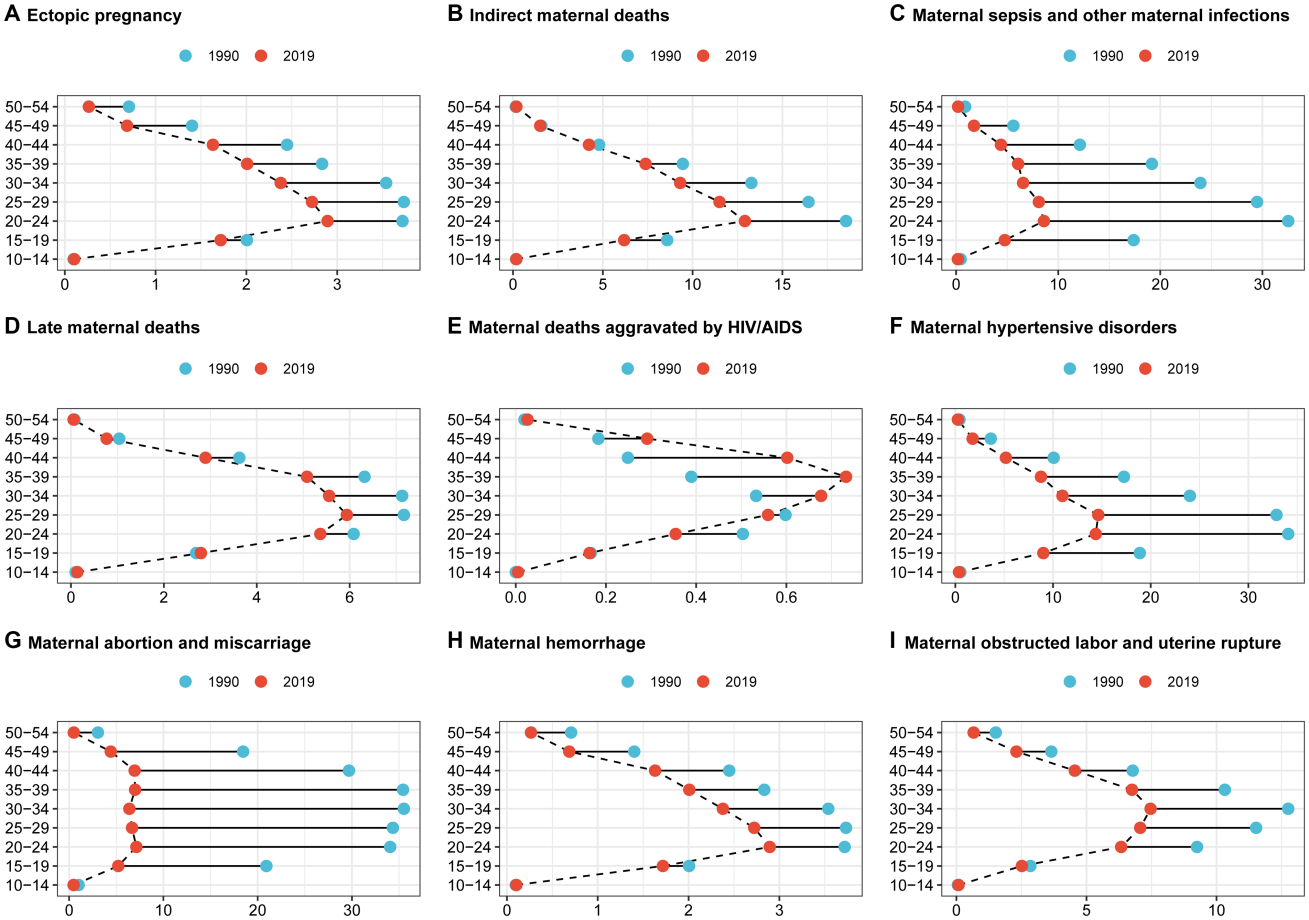
Contributions of iron deficiency to the burden of maternal disorders by age group from 1990 to 2019. **(A)** Contributions of iron deficiency to the burden of ectopic pregnancy by age group from 1990 to 2019. **(B)** Contributions of iron deficiency to the burden of indirect maternal deaths by age group from 1990 to 2019. **(C)** Contributions of iron deficiency to the burden of maternal sepsis and other maternal infections by age group from 1990 to 2019. **(D)** Contributions of iron deficiency to the burden of late maternal deaths by age group from 1990 to 2019. **(E)** Contributions of iron deficiency to the burden of maternal deaths aggravated by HIV/AIDS by age group from 1990 to 2019. **(F)** Contributions of iron deficiency to the burden of maternal hypertensive disorders by age group from 1990 to 2019. **(G)** Contributions of iron deficiency to the burden of maternal abortion and miscarriage by age group from 1990 to 2019. **(H)** Contributions of iron deficiency to the burden of maternal hemorrhage by age group from 1990 to 2019. **(I)**Contributions of iron deficiency to the burden of maternal obstructed labor and uterine rupture by age group from 1990 to 2019.

Between 1990 and 2019, the DALY rates of maternal disorders caused by iron deficiency decreased among all age groups, except for maternal deaths aggravated by HIV/AIDS, which had the highest rate in the 35–39 age group in 2019. In the 30–49 age group, the DALY rates in 2019 exceeded those in 1990 in maternal deaths aggravated by HIV/AIDS (Figure 4E). The 20–24 age group had the highest DALY rates in ectopic pregnancy, indirect maternal deaths, maternal sepsis, other maternal infections, and maternal hemorrhage (Figures 4A,B,C,H). Late maternal deaths and maternal hypertensive disorders had the highest DALY rates in the 25–29 age group, while maternal obstructed labor and uterine rupture had the highest DALY rates in the 30–34 age group. Maternal abortion and miscarriage had relatively high rates across the 20–44 age range (Figures 4D,F,I). Moreover, in 1990, the DALY rates of maternal abortion and miscarriage were highest in the 15–49 age group, and this trend continued with the most significant decline observed in 2019 (Figure 4G). This decrease was followed by maternal sepsis and other maternal infections (Figure 4C). Additionally, maternal hypertensive disorder had higher DALY rates in the 15–39 age group compared to other maternal disorders in 2019 (Figure 4F).

## 4 Discussion

In this study, we examined the global burden and trends of maternal disorders related to iron deficiency during the past 30 years. We also explored the sociodemographic factors associated with burden attributable to iron deficiency. Our study shows that from 1990 to 2019, global data indicates clear downward trends in ASDR, age-standardized DALYs, and age-standardized YLLs due to iron deficiency in maternal hemorrhage, abortion, miscarriage, hypertensive disorders, sepsis, and other maternal infections. Maternal hemorrhage had the most significant decline in disease burden, while other maternal disorders remained mostly stable, with some showing minimal change or lacking data. Regionally, mortality rates and years of life lost due to indirect maternal deaths, late maternal deaths, and deaths aggravated by HIV/AIDS increased in high SDI regions, especially in North America. At the country level, the Maldives has garnered attention due to a significant decreasing trend in age-standardized DALYs attributable to iron-related maternal hypertensive disorders and maternal hemorrhage, with other iron-related maternal conditions also demonstrating substantial reductions.

Ectopic pregnancy happens when a fertilized egg implants outside the normal uterine cavity, posing a life-threatening risk to women of reproductive age (32). Severe iron deficiency greatly impacts fertility and could be a key factor in cases of unexplained infertility (33). In the UK, it is the main cause of first-trimester pregnancy-related deaths (0.35 out of 1,000 ectopic pregnancies) (34), while in developing countries, an estimated 10% of women diagnosed with ectopic pregnancy die from the condition (35). According to the GBD 2019, iron deficiency is a major risk factor for Ectopic pregnancy. The study found that over 20% of Ectopic pregnancy deaths and DALYs in sub-Saharan Africa were due to iron deficiency. In high SDI regions, more than 10% of Ectopic pregnancy -related deaths and DALYs were linked to iron deficiency. This finding suggests new research directions for Ectopic pregnancy. Further research is needed to confirm if iron deficiency or other micronutrient deficiencies contribute to Ectopic pregnancy development (36). An indirect maternal death is when a woman dies due to pre-existing or pregnancy-related diseases that aren’t directly caused by childbirth but are made worse by pregnancy (37). According to the World Health Organization, about 28% of maternal deaths from 2003 to 2009 were from these indirect causes (38). These types of causes are known to account for many maternal deaths in high-income countries (39, 40). In low- to middle-income countries, iron deficiency anemia is one of the most common conditions associated with indirect maternal mortality (41). Most countries consider deaths within 42 days postpartum as late maternal deaths, because it’s believed that deaths during pregnancy or shortly after giving birth can be prevented. According to the ICD definition, deaths within a year postpartum due to pregnancy or exacerbated illnesses by pregnancy are included, except for accidental deaths (42). Although it’s thought that the global rate of late maternal deaths and deaths from obstetric sequelae is decreasing to 1–2% of the deaths coded with the ICD-10, recent studies have found that this percentage is increasing in the Americas (43, 44). Miscarriage is a frequent result of pregnancy, with the majority of research indicating a 12–15% rate of loss in recognized pregnancies by 20 weeks of gestation (45). A recent study found that iron deficiency may play a significant role in anemia-related miscarriage due to its association with hypoxia, oxidative stress, and increased susceptibility to infections (46). Maternal hemorrhage remains one of the top three causes of maternal deaths, with the majority of such fatalities occurring within 24–48 h after childbirth. Although the global maternal mortality rate has been steadily decreasing, it still poses a challenging issue for healthcare systems around the world (47). Maternal hypertensive disorders include chronic hypertension, gestational hypertension, and pre-eclampsia (48). These conditions complicate 2–3% of pregnancies (49). Andrew et al. concluded that perinatal iron deficiency leads to long-term, sex-dependent changes in renal metabolic function and morphology. These alterations may contribute to hypertension and an elevated risk of cardiovascular disease (50). Uterine rupture can cause serious short-term complications including severe anemia, shock, and bladder rupture. Women who survive may face long-term complications, such as vesicovaginal fistula, foot drop, and infertility due to hysterectomy or tubal ligation, the incidence rate is 0.17–0.2% (51). Obstructed labor occurs when the presenting part of the fetus cannot pass through the pelvis due to blockages at the entrance to the pelvis, within its cavity, or at the exit, even if accompanied by strong uterine contractions (52). Obstructed labor is a preventable obstetric issue (53). We all know that iron deficiency anemia can exacerbate the ischemic state of postpartum uterine rupture, increasing maternal mortality (54). Maternal sepsis is a potentially life-threatening condition during pregnancy, childbirth, or the postpartum period. According to the WHO Global Maternal Sepsis Study Research Group, around 70 out of 1,000 pregnant or recently pregnant women required hospital care due to maternal infections, and more than half of the deaths occurring in hospitals in 2017 were related to these infections (55). Lotta et al. identified that iron deficiency increased rates of prematurity, fetal growth restriction, postpartum infections and adding extra strain on families and the healthcare system (56).

Our findings, based on global data spanning from 1990 to 2019, indicate a significant decline in ASDR, age-standardized DALYs, and YLLs for maternal conditions including maternal hemorrhage, miscarriage and abortion, maternal hypertensive disorders, as well as maternal sepsis and other maternal infections, attributed to iron deficiency. Although the global targets set by the WHO aimed to reduce the prevalence of anemia in women of reproductive age by half by 2025 compared to 2010 levels, achieving this goal has fallen short of expectations. Nonetheless, the effectiveness of interventions is apparent (57). The burden of maternal hemorrhage disease showed the highest decrease, with approximately 24% of maternal disorders attributed to maternal hemorrhage according to relevant studies from the GBD 2019 (17). Previous studies have also indicated that iron deficiency in pregnant women is a significant risk factor for postpartum hemorrhage (58). This could be the primary reason for the most significant decline in maternal hemorrhage due to iron deficiency.

HIV/AIDS infection has been linked to lower Hb levels in some studies and could increase the impact of iron deficits on mortality risk (59). It’s noteworthy that the trend of age-standardized DALYs and YLLs from indirect maternal deaths, late maternal deaths, and maternal deaths aggravated by HIV/AIDS related to iron deficiency has been increasing in the high SDI region, particularly in High-income North America. Research utilizing National Vital Statistics data of the United States from 1999 to 2017 reveals a concerning pattern (60). While there has been a decrease in maternal mortality attributed to direct obstetric causes over the past two decades, there has been a significant rise in maternal deaths resulting from indirect causes, especially those related to pre-existing medical conditions such as cardiovascular disease (CVD) and metabolic disorders. The annual rates of these deaths have surged by 11.2% for the general population during this period (60). In recent years, the proportion of late maternal deaths and deaths from sequelae of obstetric causes has increased in the Americas. From 2006 to 2013 compared to 1999 to 2005, the risk of these deaths increased by 2.4 times. This confirms that late maternal deaths are more frequently recorded in death certificates in the countries included in this study, possibly due to modifications in death certificates to improve capturing of deaths occurring after 42 days postpartum, or because women are surviving longer due to improvements in pregnancy-related healthcare (44). According to findings from the third National Health and Nutrition Examination Survey (1988–1994), about 7.8 million women were identified with iron deficiency, with approximately 3.3 million of them suffering from iron deficiency anemia. Data from NHANES 2017–2018 revealed a prevalence of 48.17% (95% CI 36.84–59.69) for iron deficiency anemia and 12.08% (95% CI 8.16–17.53) for anemia (61). The burden of these diseases may be escalating, along with the burden caused by iron deficiency. This could reflect how, in these regions, even with relatively high resource conditions, iron deficiency and associated maternal health issues remain significant public health challenges.

It’s worth noting that the burden of maternal deaths aggravated by HIV/AIDS due to iron deficiency is showing a significant increase. Globally, and across different levels of SDI, the proportion of maternal deaths aggravated by HIV/AIDS is on the rise, particularly in Southern Sub-Saharan Africa, Oceania, and the region of Georgia. When examining the changes in the disease burden of maternal deaths caused by iron deficiency between 1990 and 2019, it’s evident that the DALY rates for maternal deaths aggravated by HIV/AIDS, especially in the 35–39 age group, reached their highest rate in 2019. This discovery highlights the critical importance of considering HIV/AIDS factors in iron deficiency treatment and prevention measures. In 2016, United Nations program for HIV/AIDS (UNAIDS) estimated over 35 million HIV cases worldwide. Most cases were from eastern and southern Africa (19 million), followed by western and central Africa (6.5 million), Asia and the Pacific (5.1 million), Western and central Europe, and North America (2.4 million), Latin America and the Caribbean (2 million), eastern Europe and central Asia (1.5 million), and the Middle East and northern Africa (230,000) (62). While the prevalence of HIV/AIDS has decreased in recent decades, it remains a significant public health concern in Asia and the Pacific region and, with 300,000 new HIV infection, and 150,000 deaths reported in 2022. Although there is no cure, prevention is possible (63). In regions such as Africa and the Pacific where resources are limited, these challenges may exacerbate iron deficiency-related issues (64). Although Georgia has a relatively low HIV prevalence rate (0.07%), the incidence of HIV in Georgia has been rising since the mid-1990s. This is attributed to the increasing HIV incidence in neighboring countries and several risk factors for HIV/AIDS, including a high prevalence of sexually transmitted infections, injection drug use, and limited awareness about HIV among the Georgian population (65). In addition, as a result of surveillance system functioning (2016–2019), 57% pregnant women were iron deficient (66). Our research findings indicate a worsening trend in maternal mortality exacerbated by HIV/AIDS, highlighting the need for increased consideration of HIV/AIDS factors in iron deficiency treatment and prevention measures, especially in regions like Sub-Saharan Africa and the Oceania.

Among 204 countries, we observed a significant decreasing trend in age-standardized DALYs attributable to iron-related maternal disorders in the Maldives, encompassing a range of conditions. The most notable declines were seen in maternal hypertensive disorders and maternal hemorrhage, with other iron-related maternal disorders also showing substantial reductions. These include ectopic pregnancy, indirect maternal deaths, late maternal deaths, maternal abortion and miscarriage, maternal deaths exacerbated by HIV/AIDS, maternal sepsis and other infections, and various other maternal disorders. These findings highlight the effectiveness of the Maldives’ management strategies for iron-related maternal disorders. In the Maldives, there was no comprehensive sexual and reproductive health policy or strategy until 2014. However, in 2014, the Ministry of Health, with support from the World Health Organization (WHO) and the United Nations Population Fund (UNFPA), developed the third National Reproductive Health Strategy for the period of 2014–2018. This strategy includes important aspects such as family planning, maternal health, prevention of unsafe abortion, prevention, and management of sexually transmitted infections (STIs) including HIV, and promotion of sexual health. In addition to the National Reproductive Health Strategy spanning from 2014 to 2018, international agencies in the Maldives are also carrying out key strategies such as the World Health Organization’s country cooperation strategy covering the period of 2013–2017 and the United Nations Development Assistance Framework for Maldives from 2011 to 2015. These strategies emphasize prevention as the main approach to tackling reproductive health issues (67). Based on our findings, it is evident that the burden of iron-related maternal disorders in the Maldives has been significantly reduced, indicating a notable decrease. This demonstrates the effectiveness of these measures.

Our study found a significant decrease in the DALY rates for miscarriages and preterm births among women aged 15–49. Additionally, maternal hypertensive disorders had the highest DALY rates among maternal conditions in the 15–39 age group in 2019, with the most notable reductions in DALY rates for miscarriages and abortions occurring in the 15–49 age group. For those aged 20–24, we observed the highest DALY rates for ectopic pregnancies, indirect maternal deaths, maternal sepsis, other maternal infections, and maternal hemorrhage, highlighting potential health risks and nutritional support gaps during early pregnancy. Ages 25–29 showed the highest DALY rates for late maternal deaths and maternal hypertension, while the 30–34 age group faced the highest rates for obstructive labor and uterine rupture, indicating that complications increase with maternal age. Our findings suggest that iron deficiency leads to varying burdens of maternal disorders across different age groups, underscoring the need for targeted prevention and management. According to research conducted by R. Peng et al. in 2024, although the ASRs for the incidence and prevalence of maternal disorders were highest among those aged 20–29, ASRs for mortality and DALYs due to maternal disorders were most significant in the 20–39 age group. This indicates that while these conditions may affect women across a broad age range during their reproductive years, the impact and potential for improvement in outcomes are particularly notable in specific age groups (17).

Currently, many countries are implementing policies and measures to address iron deficiency, leading to improvements worldwide. For example, India introduced a National Non-Communicable Diseases Monitoring Framework and Action Plan in 2014 to enhance youth health (68). The Global Alliance for Improved Nutrition initiated projects in 14 countries to boost iron intake, such as fortifying fish sauce in Vietnam and wheat and maize flour in South Africa (69). In 2019, China launched the “Healthy China” action plan, aiming to reduce maternal mortality by 2030 (70). In various low-income countries, the United Nations oversees iron supplementation programs, particularly for high-risk groups like pregnant women (64).

Taken together, in this research, we conduct a systematic analysis of the modeled burden of maternal disorders resulting from iron deficiency at global, regional, and national levels. Using publicly accessible data from the GBD 2019 Study, our analysis spans various age groups, sexes, and SDI categories. This approach yields the latest, most comprehensive, and comparable insights into the global impact of maternal disorders caused by iron deficiency. Our study reveals that in high SDI regions, there is an alarming increase in mortality rates and years of life lost due to indirect maternal deaths, late maternal deaths, and deaths exacerbated by HIV/AIDS related to iron deficiency. These regions should prioritize the enhanced management of these conditions and improve iron intake. Specifically, in Southern Sub-Saharan Africa, Oceania, and Georgia, there should be a particular focus on reducing deaths aggravated by HIV/AIDS related to iron deficiency.

This study has several limitations. Firstly, caution must be exercised when conducting international comparisons of disease burden due to significant differences in the quality of data on mortality, and DALYs among countries. Secondly, it’s challenging to accurately quantify and eliminate all variations caused by measurement errors when estimating epidemiological indicators. Therefore, there are discrepancies in the data results. Thirdly, due to data quality issues in low-income countries and a lack of studies reporting population-representative estimates for disease indicators, generalized assumptions had to be made, particularly regarding maternal disorders. To progress from estimations and assumptions to more certain conclusions, future research should establish specialized databases. Specifically, routine surveillance, sentinel surveillance, and special surveys are urgently needed in developing countries to gather data on maternal and neonatal disorders.

## 5 Conclusion

While mortality rates and years of life lost due to iron deficiency-related maternal disorders have decreased in most regions and subtypes, there is a concerning rise in indirect maternal deaths, late maternal deaths, and deaths aggravated by HIV/AIDS in high SDI regions, particularly North America. The proportion of maternal deaths aggravated by HIV/AIDS is also rising globally, particularly in Southern Sub-Saharan Africa, Oceania, and Georgia. In the Maldives, the age-standardized DALYs for maternal disorders attributable to iron deficiency exhibited a notable decreasing trend, encompassing a range of conditions. Additionally, there has been a significant decrease in DALYs rate for miscarriages and preterm births among women aged 15–49, with hypertensive disorders posing the highest burden among women aged 15–39.

## Data Availability

The datasets presented in this study can be found in online repositories. The names of the repository/repositories and accession number(s) can be found in the article/Supplementary material.

## Footnotes

^1^ http://ghdx.healthdata.org/gbd-results-tool

## Supplementary material

SUPPLEMENTARY TABLE S1 ICD-10 code for maternal disorder.

SUPPLEMENTARY TABLE S2 The EAPC of the ASDR for maternal disorder associated with iron deficiency.

SUPPLEMENTARY TABLE S3 The EAPC of the YLDs for maternal disorder associated with iron deficiency.

SUPPLEMENTARY TABLE S4 The EAPC of the YLLs for maternal disorder associated with iron deficiency.

SUPPLEMENTARY TABLE S5 The EAPC of the DALY burden of maternal disorders caused by iron deficiency across 204 countries.
