# Supplementary material for: The global burden of maternal disorders attributable to iron deficiency related sub-disorders in 204 countries and territories: an analysis for the Global Burden of Disease study
Source: Front Public Health. 2024 Sep 5;12:1406549. doi: 10.3389/fpubh.2024.1406549 (PMC11413869; doi:10.3389/fpubh.2024.1406549)
Supplement: SUPPLEMENTARY TABLE S1 — ICD-10 code for maternal disorder. [file Table_1.DOCX]

**Table S1 Categories of causes and their operational definitions**

| Causes | ICD-10 codes |
| --- | --- |
| **Maternal disorders** |  |
| Maternal hemorrhage | O20, O44, O45, O46, O67, O72, O73 |
| Maternal sepsis and other maternal infections | O23, O85, O75.3, O86, O91 |
| Maternal hypertensive disorders | O10, O11, O12, O13, O14, O15, O16 |
| Maternal obstructed labor and uterine rupture | O64, O65, O66 |
| Maternal abortion and miscarriage | O01, O02, O03, O04, O05, O06, O07, O08 |
| Ectopic pregnancy | O00 |
| Indirect maternal deaths | O97.1 |
| Late maternal deaths | O96 |
| Maternal deaths aggravated by HIV/AIDS | O98.7 |
| Other maternal disorders | O21, O22, O24, O25, O26, O27, O28, O29, O30-O48, O60-O71, O73, O74, O75,O87-O90, O92, O94, O95, O97-O99 |
